# Supplementary material for: Classifying Circumnutation in Pea Plants via Supervised Machine Learning
Source: Plants (Basel). 2023 Feb 20;12(4):965. doi: 10.3390/plants12040965 (PMC9965265; doi:10.3390/plants12040965)
Supplement: Supplementary file 1 [file plants-12-00965-s001.zip › plants-2177623-supplementary.pdf]

Supplementary materials for

Classifying Circumnutation in Pea Plants via Supervised Machine Learning

Qiuran Wang [1,*], Tommaso Barbariol [2], Gian Antonio Susto [2], Bianca Bonato [1], Silvia Guerra [1] and Umberto Castiello [1]

[1] Department of General Psychology, University of Padova, 35132 Padova, Italy; bianca.bonato.1@phd.unipd.it (B.B.); silvia.guerra@unipd.it (S.G.); umberto.castiello@unipd.it (U.C.)
[2] Department of Information Engineering, University of Padova, 35131 Padova, Italy; tommaso.barbariol@gmail.com (T.B.); gianantonio.susto@unipd.it (G.A.S.)
* Correspondence: qiuran.wang@gmail.com or qiuran.wang@phd.unipd.it

S1. Data acquisition

For each growth chamber, a pair of RGB-infrared cameras (IP 2.1 Mpx outdoor varifocal IR 1080P) were placed 110 cm above the ground, spaced at 45 cm to record stereo images of the plant. The cameras were connected via Ethernet cables to a 10-port wireless router (D-link Dsr-250n) connected via Wi-Fi to a PC. The frame acquisition and saving process were controlled by a CamRecorder software (Ab.Acus s.r.l., Milan, Italy). Each camera's intrinsic, extrinsic, and lens distortion parameters were estimated using a Matlab Camera Calibrator App. Depth extraction from the single images was carried out by taking 20 pictures of a chessboard (squares' size 18 × 18 mm, 10 columns × 7 rows) from multiple angles and distances in natural non-direct light conditions. For stereo calibration, the same chessboard used for the single-camera calibration process was placed in the middle of the growth chamber.

S2. Data processing

The two cameras synchronously acquired the frame every 180 seconds (frequency 0.0056 Hz). The anatomical landmarks of interest were the "tendrils" and the "junction" (i.e., the point where the tendrils tie together), developing from the considered leaf. We considered the initial frame as the one corresponding to the appearance of the tendrils and the junction of the considered leaf. The end frame was defined as the frame in which the tendrils start to coil the support for the "support" condition, or the frame just before the plant fell on the ground for the "no support" condition. An ad hoc software (Ab.Acus s.r.l., Milan, Italy) developed in Matlab was used to position post-hoc virtual markers on the tendrils and the junction to track their position frame-by-frame on the images acquired by the two cameras to reconstruct the 3D trajectory for each marker. The tracking procedures were first performed automatically throughout the time course

of the movement sequence using the Kanade-Lucas-Tomasi (KLT) algorithm on the frames acquired by each camera after distortion removal. Then, the tracking was manually verified by the experimenter, who checked the position of the tendrils and the junction frame-by-frame.

S3. Feature extraction

Features are extracted by:

- Junction trajectory (a) and tendril trajectory (b): 3D trajectories for the junction and the tendrils were acquired in Cartesian coordinates (x, y, z), where x and y axis form the vertical dimension, and x and z form the horizontal dimension. The coordinates for the tendrils are termed as (x_t, y_t, z_t), and those for the junction as (x_n, y_n, z_n).
- Junction velocity (c); tendril velocity (d): the velocity of the junction was calculated by computing the absolute value between the difference with n_i frames and n_{i+1} frames (i: 1, 2, 3, …, n). The velocity of the tendrils and the junction for each axis (v_x, v_y, v_z) and for each frame were acquired.
- Junction acceleration (e) and tendril acceleration (f): acceleration for the junction and the tendrils was calculated as a velocity derivative.
- Tendril aperture (g): relative vectors from the junction to the tip of the tendrils were extracted by calculating the mean of the tendrils ($\overline{X}_t, \overline{Y}_t, \overline{Z}_t$), minus the coordinates of the junction (x_n, y_n, z_n). Depending on the number of tendrils that one plant possesses, the tendril number could be either two or three. The standard deviation of the tendrils (σ_x, σ_y, σ_z), indicates the variability of the tendrils' aperture.
- Overall movement duration (h).
- Movement duration for single circumnutations (i).

On the basis of this the features considered for model classifications were: (a) junction trajectory; (b) tendril trajectory; (c) junction velocity; (d) tendril velocity; (e) junction acceleration; (f) tendril acceleration; (g) tendrils aperture; (h) overall movement duration; (i) movement duration for single each circumnutation; (j) all features (i.e., the full kinematic picture).

S4. Classifiers

Random Forests classifier (RF) is a method of conjoint learning for classification and regression which operates by building many decision trees during training and generating the class of individual trees [1]. The decision forests correct the trend of the trees in adjusting to their training dataset. A random forest is a meta estimator that fits several decision tree classifiers on various sub-samples of the dataset and uses averaging to improve the predictive accuracy and is robust against over-fitting [1,2].

Logistic Regression (RF) is a machine learning classification algorithm that calculates the class membership probability for one of the two categories in the data set [3]. It assumes binary logistic regression and requires the dependent variable to be a binary category.

The Support Vector Classification (SVC) derives from the SVM (Support Vector Machine) and is a model of supervised associated learning, used for classification and regression analysis. An SVM training algorithm builds a model that attributes new examples to one or another category, making it a linear non-probabilistic binary classifier [4].

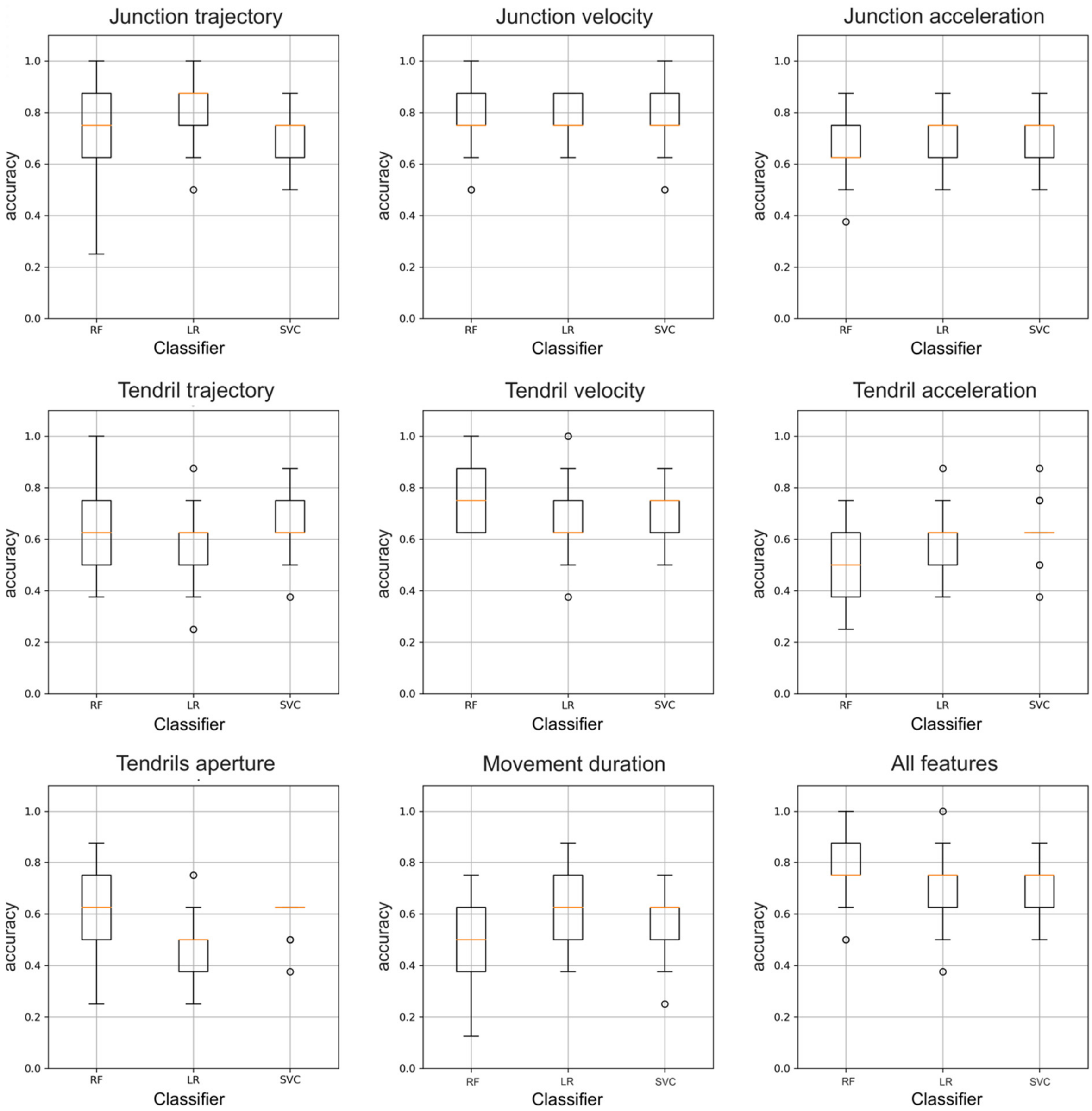

Figure S1. Specific contribution of the considered features across classifiers for the overall movement classification.

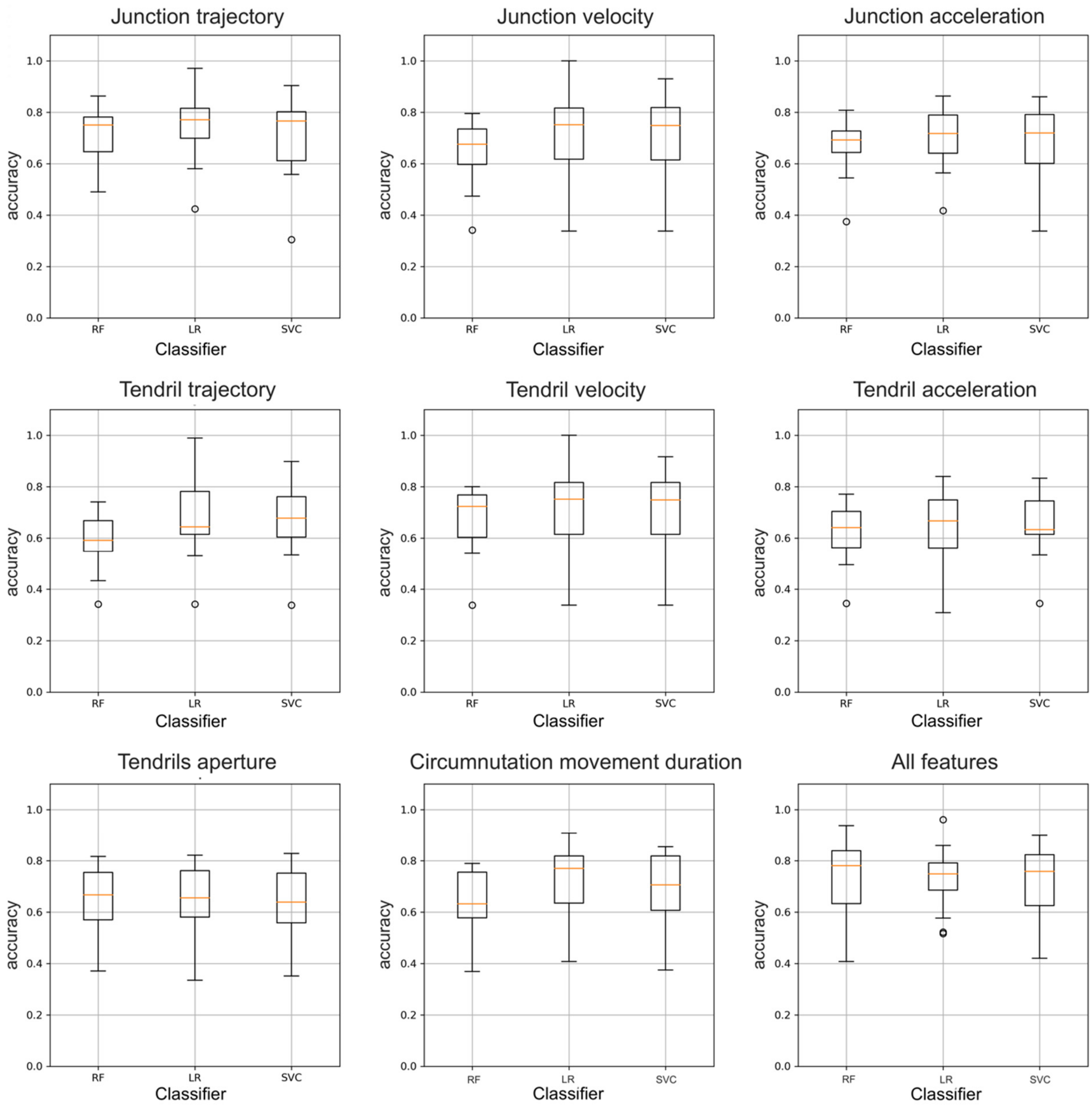

Figure S2. Specific contribution of the considered features when considering single circumnutation.

References

1. Breiman, L. Random forests. *Machine Learning* **2001**, *45*, 5-32, doi:https://doi.org/10.1023/a:1010933404324.
2. Liaw, A.; Wiener, M. Classification and regression by randomForest. *R News* **2002**, *2*, 18-22.
3. Dreiseitl, S.; Ohno-Machado, L. Logistic regression and artificial neural network classification models: A methodology review. *Journal of Biomedical Informatics* **2002**, *35*, 352-359, doi:doi.org/10.1016/S1532-0464(03)00034-0.
4. Manevitz, L.M.; Yousef, M. One-class SVMs for document classification. *Journal of Machine Learning Research* **2001**, *2*, 139-154.
